# Characterization and comparison of bacterial communities in benign vocal fold lesions

**DOI:** 10.1186/2049-2618-2-43

**Published:** 2014-12-08

**Authors:** Alissa S Hanshew, Marie E Jetté, Susan L Thibeault

**Affiliations:** Department of Surgery, University of Wisconsin, 1111 Highland Avenue, Madison, 53705 Wisconsin USA

**Keywords:** Larynx, Voice disorders, Benign lesions, Microbiota, *Streptococcus*

## Abstract

**Background:**

Benign vocal fold lesions, including cysts, nodules, polyps, and Reinke’s edema, are common causes of hoarseness and subsequent voice disorders. Given the prevalence of these lesions, disease etiology and pathophysiology remain unclear and their microbiota has not been studied to date secondary to the paucity of available biopsies for investigation. We sought to characterize and compare the bacterial communities in biopsies of cysts, nodules, polyps, and Reinke’s edema collected from patients in Germany and Wisconsin. These samples were then compared to the communities found in healthy saliva and throat samples from the Human Microbiome Project (HMP).

**Results:**

454 pyrosequencing of the V3–V5 regions of the 16S rRNA gene revealed five phyla that explained most of the bacterial diversity, including *Firmicutes* (73.8%), *Proteobacteria* (12.7%), *Bacteroidetes* (9.2%), *Actinobacteria* (2.1%), and *Fusobacteria* (1.9%). Every lesion sample, regardless of diagnosis, had operational taxonomic units (OTUs) identified as *Streptococcus*, with a mean abundance of 68.7%. Most of the lesions, 31 out of 44, were indistinguishable in a principal coordinates analysis (PCoA) due to dominance by OTUs phylogenetically similar to *Streptococcus pseudopneumoniae*. Thirteen lesions not dominated by *S. pseudopneumoniae* were more similar to HMP throat and saliva samples, though 12 of them contained *Pseudomonas*, which was not present in any of the HMP samples. Community structure and abundance could not be correlated with lesion diagnosis or any other documented patient factor, including age, sex, or country of origin.

**Conclusions:**

Dominance by *S. pseudopneumoniae* could be a factor in disease etiology, as could the presence of *Pseudomonas* in some samples. Likewise, decreased diversity, as compared to healthy saliva and throat samples, may be associated with disease, similar to disease models in other mucosal sites.

**Electronic supplementary material:**

The online version of this article (doi:10.1186/2049-2618-2-43) contains supplementary material, which is available to authorized users.

## Background

At any one time, an estimated 20.7 million people in the US report problems with their voice, while 93.8 million report having problems during their lifetime [1]. Additionally, an estimated 22.6 million of the general population reports missing one or more days of work annually because of voice problems [1]. Voice problems are also associated with negative effects on quality of life including impaired communication, social isolation, and decreased occupational productivity [2–4]. Vocal folds play a key role in voice production and protection of the lungs, vibrating to produce sound and closing to protect the lungs against food and liquid aspiration during swallowing. Tissue of the vocal folds is unique and complex, composed of an epithelial layer made up of non-keratinized stratified squamous epithelium undergoing constant regeneration, which is bound to a three-layered lamina propria via basement membrane zone anchoring fibers. Vocal folds are also immunologically active serving both as a physical barrier and as a site that may detect early shifts in microbial presence [5].

While voice problems are associated with many causes, benign vocal fold lesions are one of the most frequent medical diagnoses [6]. Benign vocal fold lesions are generally classified into two broad categories: non-neoplastic lesions and neoplastic lesions. Non-neoplastic vocal fold lesions make up the majority of benign lesions and include cysts, nodules, polyps, Reinke’s edema, granulomas, ectasias, sulcus vocalis, and scar. Benign vocal fold lesions are typically distinguished by their phenotype, but there is limited knowledge of the etiology and progression of these diseases [7]. Mechanical, chemical, and thermal trauma; overuse and misuse; or combinations of these factors are thought to contribute to lesion development via remodeling of the lamina propria. Studies investigating histological differences and gene expression profiles have demonstrated differential stages of wound maturation and disordered wound healing [8–10]. While inflammation has historically been associated with polyps, Kotby et al. suggests that polyps and Reinke’s edema may represent a continuum of vocal fold injury [11]. Many comorbidities have been associated with vocal fold lesions, including smoking, laryngopharyngeal reflux, infection, and allergies [12–14]. To date, microbial contributions to the etiology of vocal fold lesions have focused mostly on microbes stereotypically considered pathogens, such as human papillomavirus and *Mycobacterium tuberculosis* [15–17]. However, there remains a lack of data in the literature regarding microbial community membership for normal and diseased larynx [18], secondary to a paucity of available tissue for study.

The only study published to date on laryngeal bacterial communities suggested that laryngeal squamous cell carcinoma (LSCC) could be associated with shifts in bacterial communities in the larynx [19]. Changes in the abundance of 15 genera were associated with carcinoma versus control patients with vocal fold polyps. They postulated that increases in *Fusobacterium* and *Prevotella* might be involved in initiating biofilm formation and that it could contribute to disease progression. Similar work in other mucosal sites also suggests that changes in bacterial community membership and abundance, despite the absence of classically defined pathogens, may be associated with disease. Asthmatics have been found to have altered microbial communities in their lungs [20]. While the community members responsible for this change varied between patients, a shift towards Gram negative-dominated communities was associated with elevated lipopolysaccharide levels. The authors postulated that this may be associated with increased airway cell stimulation contributing to disease. Likewise, Wang *et al*. found that the abundance of particular operational taxonomic units (OTUs) were shifted in feces from patients with colorectal cancer, particularly a reduction in butyrate-producing bacteria from the family *Lachnospiraceae* [21]. They surmised that an increase in potential pathogens coupled with a decrease in butyrate-producing bacteria could play a role in tumor formation or contribute to colorectal cancer formation.

Shifts in bacterial communities have been associated with diseases of mucosal surfaces, and we postulated that similar changes might be found in benign vocal fold lesions. In this investigation we characterized and compared the bacterial communities of benign vocal fold lesions, including biopsies of cysts, nodules, polyps, and Reinke’s edema. We hypothesized that distinct lesion types would be represented by distinct bacterial communities. This was achieved with 454 pyrosequencing of the V3-V5 region of the 16S rRNA gene using lesion biopsies in patients from the state of Wisconsin, USA, and Germany.

## Results

### Clinical diagnoses and patient characteristics

Forty-nine patients diagnosed with benign vocal fold lesions were enrolled in this study. Forty-four lesions in total were collected and yielded sufficient sequences, including 7 cysts, 5 nodules, 18 polyps, and 14 Reinke’s edema (Table 1). Characteristics of these patients are detailed in Table 1, including mean age, gender, and country of origin. Five of the 49 initial samples yielded less than 500 sequences; these samples were eliminated from further analyses.

**Table 1:** **Demographic and characteristics of participants**

	Cysts	Nodules	Polyps	Reinke’s edema
Total number of participants	7	5	18	14
Mean age (range)	57 (37–73)	31 (23–38)	44 (24–77)	52 (27–74)
Female/male	6/1	5/0	8/10	14/0
Wisconsin/German	6/1	0/5	6/12	2/12

The table includes information only for samples that resulted in more than 500 sequences and were included in further analyses.

### Bacterial communities associated with benign vocal fold lesions

Three 454 GS Junior runs resulted in a total of 248,579 high-quality sequences with a mean length of 471 bp after quality filtering and removal of primers and adapter sequences. A total of 44 samples were successfully pyrosequenced and yielded over 500 sequences. Samples had a mean of 5,650 sequences (range 892–23,996). The lowest Good’s coverage was 0.979 suggesting that communities were well sampled. No significant differences were detected between the lesion types by Chao, inverse Simpson, or Shannon diversity indexes (Table 2).

**Table 2:** **Mean sequence richness, evenness, and diversity in lesion samples**

	Cysts	Nodules	Polyps	Reinke’s edema
Chao	105.2 ± 25.7	191.0 ± 97.0	112.5 ± 17.4	111.3 ± 15.5
1/Simpson	7.4 ± 2.9	6.1 ± 2.8	4.0 ± 1.3	4.1 ± 1.5
Shannon	1.9 ± 0.5	1.8 ± 0.6	1.3 ± 0.3	1.3 ± 0.3
Good’s coverage	0.991 ± 0.002	0.996 ± 0.001	0.994 ± 0.001	0.991 ± 0.001

Mean Good’s coverage, Chao, inverse Simpson, and Shannon are reported for each of the four lesion types, along with standard error. Values for Chao, Inverse Simpson, and Shannon were not significantly different.

**Figure 1:**
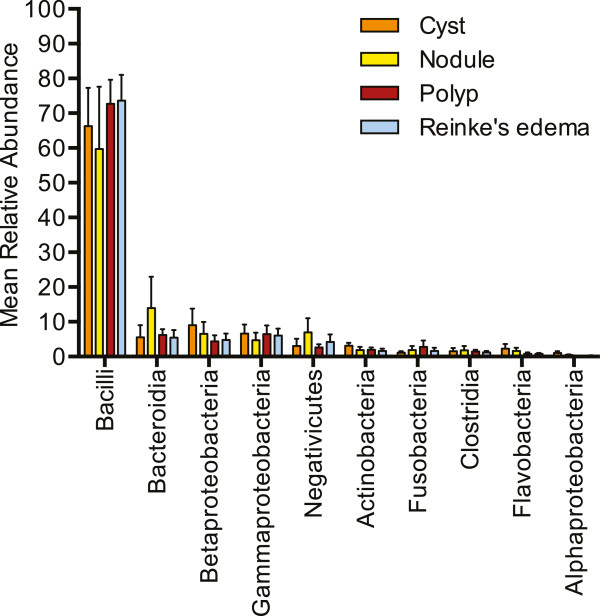
**Taxonomic composition in benign vocal fold lesions.** Mean relative abundance of the ten most common and abundant bacterial classes in cysts, nodules, polyps, and Reinke’s edema. Error bars represent standard error.

After removing rare sequences, those present at less than 0.1% abundance, 334 OTUs were present at 97% similarity across all samples, representing 5 bacterial phyla, including, in order of mean abundance, *Firmicutes* (73.8%), *Proteobacteria* (12.7%), *Bacteroidetes* (9.2%), *Actinobacteria* (2.1%), and *Fusobacteria* (1.9%). Sequences identified as *Spirochaetes*, *Cyanobacteria*, *Verrucomicrobia*, *Deinococcus-Thermus*, *Tenericutes*, and unclassified were also present, but inconsistently and at low abundance (mean less than 0.2%). Individual samples had a mean of 31.6 OTUs, and only ten classes of bacteria explained most of the diversity found, including *Actinobacteria*, *Bacilli*, *Bacteroidia*, *Clostridia*, *Flavobacteria*, *Fusobacteria*, *Negativicutes*, and *Alpha*-*, Beta-*, and *Gamma-proteobacteria* (Figure 1). Most of the sequences identified as *Bacilli* belonged to the genus *Streptococcus.* This genus was the most common and abundant, found in every lesion sample. An average of 68.7% of all sequences were identified as *Streptococcus* (cyst, 60.3%; nodule, 58.1%; polyp, 71.5%; and Reinke’s edema, 72.9%). Many samples, 31 out of 44, were dominated (at least 50% of total sequences) by OTUs identified as *Streptococcus*. Sequences identified as *Helicobacter* were present in five samples, one from Wisconsin, and four from Germany, between 0.03%–1% relative abundance.

**Figure 2:**
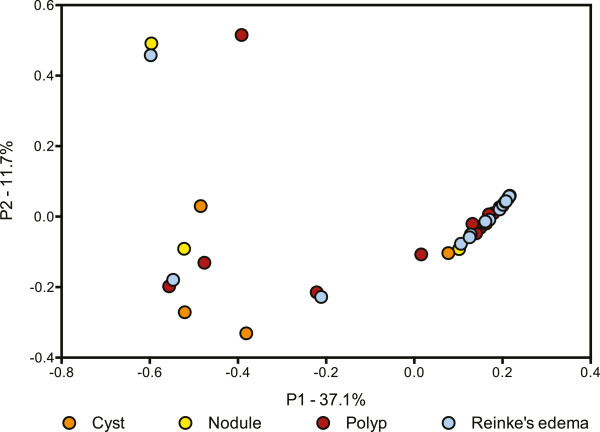
**Comparison of bacterial community structure in benign vocal fold lesions.** PCoA based on Theta Yue Clayton distances illustrating the clustering of most lesion samples, regardless of lesion diagnosis. Lesion samples are shown as circles, cysts as orange circles, nodules as yellow circles, polyps as red circles, and Reinke’s edema as blue circles.

Bacterial community comparison of individual samples supported the above observations. Communities did not cluster in the principal coordinates analysis (PCoA) plot based on lesion type (Figure 2), gender, country of origin, or age (data not shown), nor any other documented clinical information. Dominance by *Streptococcus* is evident in Figure 2, where the 31 samples dominated by *Streptococcus* all cluster together.

### Comparison of healthy HMP samples to lesion communities

Due to the unethical nature of taking biopsies from healthy human vocal folds, for further analyses we included 15 randomly selected saliva and throat samples from the Human Microbiome Project (HMP) as proxies for healthy vocal fold bacterial communities. Saliva was the most diverse sample type and was significantly different from all other samples by both inverse Simpson and Shannon (*p* < 0.0001) (Table 3). Throat samples were intermediary, and the differences in diversity compared to lesion samples were not always statistically supported.

**Table 3:** **Mean sequence richness, evenness, and diversity in lesion and HMP samples**

	Cysts	Nodules	Polyps	Reinke’s edema	Saliva	Throat
Chao	96.9 ± 23.4^b^	121.7 ± 43.5	98.1 ± 15.6^c^	90.2 ± 11.6^a^	190.1 ± 12.2^abc^	140.6 ± 11.5
1/Simpson	7.4 ± 2.9	6.0 ± 2.8	4.0 ± 1.3^e^	4.1 ± 1.5^d^	17.7 ± 1.6^*^	11.0 ± 1.4^de^
Shannon	1.9 ± 0.5	1.8 ± 0.6	1.3 ± 0.3^g^	1.3 ± 0.3^f^	3.4 ± 0.1^**^	2.8 ± 0.1^fg^
Good’s coverage	0.991 ± 0.002	0.997 ± 0.001	0.994 ± 0.001	0.993 ± 0.001	0.988 ± 0.001	0.992 ± 0.001

Mean Good’s coverage, Chao, inverse Simpson, and Shannon are reported for each of the four lesion types, and saliva and throat samples from the HMP, along with standard error.

^*^Saliva was significantly different from all other groups for inverse Simpson (*p* < 0.0001).

^**^Saliva was significantly different from all lesion samples for Shannon, but not throat (*p* < 0.0001).

^a–g^Pairwise comparisons that were significantly different (*p* < 0.0001).

**Figure 3:**
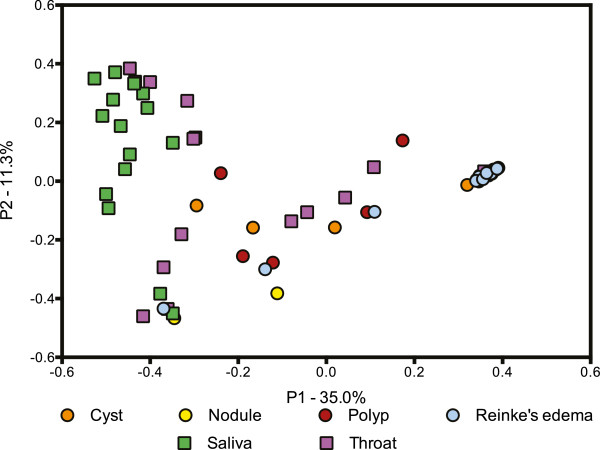
**Comparison of bacterial community structure in benign vocal fold lesions and throat and saliva samples from the HMP.** PCoA based on Theta Yue Clayton distances illustrating the clustering of most lesion samples, regardless of lesion diagnosis with the inclusion of 15 randomly selected throat and saliva HMP samples. Lesion samples are shown as circles, cysts as orange circles, nodules as yellow circles, polyps as red circles, and Reinke’s edema as blue circles. HMP samples are shown as squares, saliva as green squares, and throat as purple squares.

Tight clustering of lesion samples was not altered in the PCoA plot with the inclusion of the HMP samples (Figure 3). However, some lesions had communities more similar to saliva and throat samples than to other lesions. OTUs identified as *Pseudomonas* were present in 12 of the 13 lesions that did not cluster based on dominance by *Streptococcu*s, though *Pseudomonas* was found in only one throat sample.

### The abundance of *Streptococcus*in lesion samples

**Figure 4:**
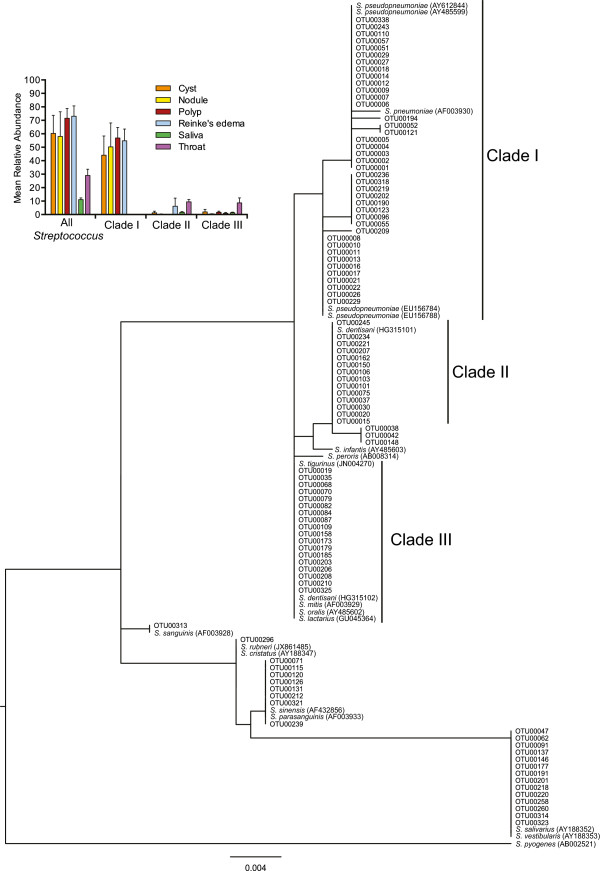
**Phylogenetic relationship and abundance of the top 100 unique** ***Streptococcus*** **OTUs.** The top 100 unique *Streptococcus* OTUs along with 21 reference *Streptococcus* sequences were phylogenetically analyzed. Three major clades were found. Sequences from clade I dominated the lesion samples while being completely absent from HMP samples. Panel inset represents the mean relative abundance of all *Streptococcus* OTUs in cysts, nodules, polyps, Reinke’s edema, saliva, and throat, in addition to the portion represented by clades I, II, and III. Error bars represent standard error.

At 97% sequence identity, one OTU identified as *Streptococcus* was present in every lesion sample at a mean abundance of 66.21% (range 0.76%–99.76%, 5.01 SE). The sequence selected by mothur to represent this OTU was identified as *S. pseudopneumoniae*. However, 16S sequences in the mitis group, of which *S. pseudopneumoniae* is a member, often have greater than 99% sequence identity [22, 23]. We used the 100 most abundant unique OTUs identified as *Streptococcus* to further explore the diversity and phylogenetic relationship of this genus present in our samples and the included HMP samples (Figure 4). Forty of the top 100 *Streptococcus* OTUs claded with *S. pneumoniae* and *S. pseudopneumoniae*, labeled as clade I, which differ by only one base pair in the region sequenced. Thirty-six of the 44 lesion samples contained OTUs in clade I, while this clade was not present in saliva or throat samples (Figure 4, panel inset). Manual inspection of the alignment file indicated that OTUs within clade I differed in only short homopolymer sequences, 2–6 bp in length. Clades II and III were less common and abundant (Figure 4, panel inset). OTUs in clade II grouped with *Streptococcus dentisani*, a recently described *Streptococcus* species [24]. Clade III OTUs could not be identified to the species level, as some species in the mitis group are indistinguishable in this region of the 16S rRNA gene [23]. OTUs that grouped outside these clades were also present, but at very low abundance (data not shown).

## Discussion

Maintenance of vocal fold function is essential for human health, where the consequences of impaired voice production hold profound implications for individual health and wellness, social and occupational function, and societal productivity [3, 4]. Benign vocal fold lesions are one of the most common diagnoses when problems with voice arise, but a full pathophysiologic understanding of how and why these lesions form is still lacking. We sought to describe the bacterial communities in a large set of vocal fold biopsies that included cysts, nodules, polyps, and Reinke’s edema. Surprisingly, the majority of our samples, 31 out of 44, had highly similar bacterial communities dominated by *S. pseudopneumoniae*, regardless of lesion type, or any other documented patient characteristic. To date, only one other study has looked at whole bacterial communities in the larynx, comparing LSCC to nearby “healthy” tissue, and a control group of patients with polyps [19]. Broad differences are apparent between their control polyp patients and ours, from the phyla level to the genus level, where we found considerably more *Streptococcus* (71% vs. 56%), but less of all other genera they deemed dominant, including *Fusobacterium* (0.6% vs. 8%), *Prevotella* (5% vs. 7%), *Neisseria* (2% vs. 5%), and *Gemella* (0.5% vs. 2%). It is widely accepted that even minor differences in sample preparation can lead to discrepancies in community composition, and the differences found between our polyp samples and those in Gong *et al.* could be due to any one of these factors, including the use of different primer pairs or DNA extraction protocols, and modifications to PCR protocols [19, 25–32].

The presence of *Streptococcus* is well noted in healthy oral and respiratory sites [33–35]. However, due to the high similarity of the 16S gene in many *Streptococcus* species, finer resolution based on non-full length sequences is difficult. A phylogeny of the OTUs identified as *Streptococcus* in our study demonstrated that many of them were either *S. pseudopneumoniae* or *S. pneumoniae*. These two *Streptococcus* species vary by a single base pair in the region sequenced here, and a manual check of the alignment showed that all sequences in clade I contained a cytosine at that position, like *S. pseudopneumoniae*, and no OTU out of the top 100 could be identified as *S. pneumoniae*. Dominance by *S. pseudopneumoniae* in the majority of our samples may be playing a role in disease progression. This recently described member of the mitis group has many genes associated with host cell interaction, including some of which are thought to be virulence factors [36, 37]. *S. pseudopneumoniae*, previously thought to be atypical *S. pneumoniae*, has been isolated from patients with a number of respiratory diseases including chronic obstructive pulmonary disease (COPD), cystic fibrosis, pneumonia, bronchitis, and chronic sinusitis [38–40]. However, its status as a pathogen, or mutualist, is still not clear. All 20 human subjects in a tonsillar crypt study were found to have *S. pseudopneumoniae*, regardless of tonsil health status [41].

*Helicobacter pylori* is perhaps one of the most prevalent “infections” in the world, estimated to colonize the stomach of over 50% of the world’s human population [42]. Work in the last few decades has shown a direct relation between *H. pylori* and stomach cancer [42], and more recently, correlations have been made with other aerodigestive tract diseases, such as otitis media with effusion [43] and oral aphthous ulcers [44]. *H. pylori* has also been associated with LSCC [45–47], though there is debate about whether it is associated with benign vocal fold lesions [48] or not [47, 49]. Only a few of the samples presented here contained *Helicobacter* and at very low abundances. Many studies seeking to tie vocal fold lesions to *H. pylori* infections have looked for the specific presence of the bacterium in the larynx [47], but not necessarily at its abundance. It could be that *H. pylori* is common in vocal folds, but at a low enough abundance that it was mostly missed in our patient population and with our sampling technique, even in those with previously diagnosed infections.

One limitation in our study design was the inability to compare these lesions to the microbial community that may be found in biopsied healthy human vocal folds. The lamina propria of the vocal folds is very thin, only 3 mm thick, and the possibility of creating vocal scar and impairing voice production is present with every surgical procedure including biopsy. As such, it is considered unethical to biopsy vocal folds of healthy human subjects. As a proxy, we included in our analyses 15 randomly selected saliva and throat samples on the basis that many of the habitats above the stomach have similar microbial communities due to the buffering nature of saliva, regular nutrient availability in the form of mucin, and a common epithelial lining (non-keratinized, stratified, squamous epithelium) [33]. Of the 15–18 body sites sampled by the HMP, non-tooth oral sites, including saliva and throat, were deemed to be highly similar [50]. Samples from these body sites might have the most in common with our samples, considering that they all face similar environmental exposures such as inhaled air, ingested substances (food and beverage), and constant contact via saliva and a continuous mucus layer. Indeed, 13 of our 44 samples were highly similar to these throat and saliva samples. However, the majority harbored a distinct community, one dominated by *S. pseudopneumoniae,* as described above. Interestingly, of the 13 samples more similar to saliva and throat, 12 of them contained *Pseudomonas*, a genus found in only 1 throat sample. However, many of the common and dominant genera found in the saliva and throat samples were also found in these 13 samples, including *Actinomyces, Prevotella, Fusobacterium, Leptotrichia, Neisseria, Haemophilus, Gemella, Granulicatella, Oribacterium, Veillonella,* unclassified *Prevotellaceae*, and unclassified *Lachnospiraceae* [33]. *Pseudomonas* has been associated with diseases of the lower respiratory system including bronchiolitis obliterans syndrome [51] and cystic fibrosis [52] and could be playing a role in benign vocal fold lesion etiology.

## Conclusions

Benign vocal fold lesions are usually diagnosed based on phenotypic differences present upon videostroboscopic examination. While histological and gene expression differences have been noted, the data presented here adds to the idea that despite phenotypic differences, benign vocal fold lesions share many similarities, including highly similar bacterial communities that are distinct from healthy throat and saliva samples [8, 9]. The possibility remains that these shifts in the bacterial community could inhibit wound healing, or that the presence of inflammation creates a niche for a community dominated by *S. pseudopneumoniae*.

## Methods

### Subjects and collection of benign lesions

Benign vocal fold lesions were collected at University of Wisconsin-Madison, USA, and the University of Hamburg, Germany. Laryngeal microsurgical techniques were used to remove lesions. For each lesion, the clinical diagnosis was made based on initial videostroboscopic exam and confirmed under direct visualization by the surgeon at the time of surgical removal. Samples were immediately placed in RNAlater (Ambion Inc., Austin, Texas) and stored at -80°C until use. The University of Wisconsin Madison Health Sciences IRB and the University of Hamburg Ethics Committee approved the protocol for attainment of all tissue samples. All subjects provided written consent to participate in this study. Meta data for each sample is detailed in Additional file 1: Table S1.

### DNA extraction and PCR for 454 pyrosequencing

DNA was extracted from tissue with the EpiCenter MasterPure Complete DNA and RNA Purification Kit (Illumina, Madison, WI) with modifications to the manufacturer’s protocol. Samples were gently thawed at room temperature and briefly centrifuged to collect tissue. RNAlater was removed by pipetting and 300 μl of tissue and cell lysis solution was added to the tube with the tissue. Lysis solution and tissue were then transferred to a sterile screw top tube containing 150–200 mg of 400 μM silica beads. Proteinase K, 100 μg, was added; the tubes were vortexed and incubated at 55°C for 1 h, with vortexing every 15 min. Bead tubes were then shaken in a horizontal adapter on the vortex for 10 min. Rnase A, 5 μg, was added; the tubes were vortexed and incubated at 37°C for 30 min. The remainder of the manufacturer’s protocol was followed as written. DNA was resuspended in TE buffer and stored at 4°C until use.

PCRs were performed in triplicates containing 50–100 ng of template DNA, 0.2 μl AccuPrime Taq DNA Polymerase (Life Technologies, Grand Island, NY), 2.5 μl Buffer II, 400 nM both forward and reverse primers, and water to 25 μl total. Thermocycling conditions were as follows: 95°C 2 min, followed by 30 cycles of 95°C 20 s, 56°C 30 s, 72°C 1 min, and a final extension of 72°C 8 min. The primers included 357 F and 926R, as suggested by the HMP [53], where 357 F contained the B adapter for 454 pyrosequencing, and 926R contained both the A adapter and a 10-base pair multiplex identifier. Triplicate PCRs were pooled and cleaned using Purelink PCR purification kit (Invitrogen, Grand Island, NY) as per manufacturer’s directions for removal of primer dimers and short PCR products <300 bp. Cleaned products were eluted in 30 μl of elution buffer. Samples were then gel extracted from a low-melt agarose gel using Zymoclean Gel DNA Recovery Kit (Zymo Research, Irvine, CA) by visualizing on a blue light transilluminator (Clare Chemical Research, Dolores, CO). Cleaned PCR products were quantified using a Qubit fluorometer (Invitrogen, Grand Island, NY). Products were diluted and pooled at equal concentrations for 454 pyrosequencing.

### 454 pyrosequencing, data analysis, and statistics

454 pyrosequencing was conducted on a Roche GS Junior (Roche, Indianapolis, IN) using titanium chemistry and long read modifications found in Hanshew *et al.* [26]. Samples were sequenced across three picotiter plates. Raw data were processed using mothur (v. 1.33.1) [54], with most of the defaults put forth in the Schloss 454 SOP (http://www.mothur.org/wiki/Schloss_SOP; accessed Jan 27, 2014) [55], but with minflows = 350 and maxflows = 720 [56]. Sequences were aligned to a Silva-derived reference data base (v. 102 as implemented for mothur) [57]. Chimeras were detected using UCHIME and removed [58]. Sequences were assigned to taxonomic groups using the Ribosomal Database Project (RDP)-derived reference database [59]. All eukaryotic and unclassifiable reads were removed after classify.seqs. Sequences were assigned to OTUs at 97% sequence identity, used to construct a distance matrix using theta Yue and Clayton values, and analyzed with PCoA plots in Prism. Good’s coverage, Chao, inverse Simpson, and Shannon were calculated in mothur. One-way ANOVA with TukeyHSD *p* value correction for pairwise comparison was used to assess differences in Chao, inverse Simpson, and Shannon. In addition to lesion diagnosis, bacterial communities were also compared based on age, gender, date of surgery, and geographic location. The data sets supporting the results of this article are available in the NCBI sequence read archive, SRP047304.

Thirty HMP samples, including 15 haphazardly selected saliva and 15 throat (oropharynx) samples, were included in further analyses as proxies for “healthy” (Additional file 1: Table S1). As described above, Good’s coverage, Chao, inverse Simpson, and Shannon diversity indexes were calculated along with theta Yue and Clayton values for PCoA at 97% sequence identity [53, 60].

Similar to Jensen *et al.* and Scholz *et al.*, the 100 most abundant unique OTUs identified as *Streptococcus* were chosen for phylogenetic analysis [41, 61]. Sequences were aligned in MEGA6 using ClustalW and trimmed at the ends [62]. A phylogenetic tree was created in MEGA6 using maximum likelihood with 500 bootstraps. Sequences were searched in the Ribosomal Database Project, and matching type strain sequences were included in the phylogenetic analysis, including *S. cristatus* (AY188347), *S. infantis* (AY485603), *S. lactarius* (GU045364), *S. mitis* (AF003929), *S. oralis* (AY485602), *S. parasanguinis* (AF003933), *S. peroris* (AB008314), *S. pneumoniae* (AF003930), *S. pseudopneumoniae* (AY612844, AY485599, EU156784, and EU156788), *S. salivarius* (AY188352), *S. sanguinis* (AF003928), *S. sinensis* (AF432856), *S. vestibularis* (AY188353) [59, 63]. Non-type strains *S. dentisani* (HG315101 and HG315102), *S. rubneri* (JX861485), and *S. tigurinus* (JN004270) were included due to the high match to some OTUs. *S. pyogenes* (AB002521) was included as an outgroup.

## Electronic supplementary material

Additional file 1: Table S1: Meta data for each lesion sample along with a list of the haphazardly chosen HMP samples. (XLSX 16 KB)

## Abbreviations

OTU: operational taxonomic units
PCoA: principal coordinates analysis
HMP: Human Microbiome Project
LSCC: laryngeal squamous cell carcinoma
COPD: chronic obstructive pulmonary disease
RDP: Ribosomal Database Project.

## References

[1] Roy N, Merrill RM, Gray SD, Smith EM (2005). Voice disorders in the general population: prevalence, risk factors, and occupational impact. Laryngoscope.

[2] Cohen SM, Dupont WD, Courey MS (2006). Quality-of-life impact of non-neoplastic voice disorders: a meta-analysis. Ann Otol Rhinol Laryngol.

[3] Jacobson BH, Johnson A, Grywalski C, Silbergleit A, Jacobson G, Benninger MS, Newman CW (1997). The voice handicap index (VHI): development and validation. Am J Speech Lang Pathol.

[4] Ma EP-M, Yiu EM-L (2001). Voice activity and participation profile: assessing the impact of voice disorders on daily activities. J Speech Lang Hear Res.

[5] Thibeault SL, Rees L, Pazmany L, Birchall MA (2009). At the crossroads: mucosal immunology of the larynx. Mucosal Immunol.

[6] Cohen SM, Kim J, Roy N, Asche C, Courey M (2012). Prevalence and causes of dysphonia in a large treatment-seeking population. Laryngoscope.

[7] Cipriani NA, Martin DE, Corey JP, Portugal L, Caballero N, Lester R, Anthony B, Taxy JB (2011). The clinicopathologic spectrum of benign mass lesions of the vocal fold due to vocal abuse. Int J Surg Pathol.

[8] Duflo SM, Thibeault SL, Li W, Smith ME, Schade G, Hess MM (2006). Differential gene expression profiling of vocal fold polyps and Reinke’s edema by complementary DNA microarray. Ann Otol Rhinol Laryngol.

[9] Thibeault SL, Gray SD, Li W, Ford C, Smith ME, Davis RK (2002). Genotypic and phenotypic expression of vocal fold polyps and Reinke’s edema: a preliminary study. Ann Otol Rhinol Laryngol.

[10] Marcotullio D, Magliulo G, Pietrunti S, Suriano M (2002). Exudative laryngeal diseases of Reinke’s space: a clinicohistopathalogical framing. J Otolaryngol.

[11] Kotby M, Nassar A, Seif E, Helal E, Saleh M (1988). Ultrastructural features of vocal fold nodules and polyps. Acta Otolaryngol.

[12] Kuhn J, Toohill RJ, Ulualp SO, Kulpa J, Hofmann C, Arndorfer R, Shaker R (1998). Pharyngeal acid reflux events in patients with vocal cord nodules. Laryngoscope.

[13] Ulualp SO, Toohill RJ, Shaker R (1999). Pharyngeal acid reflux in patients with single and multiple otolaryngologic disorders. Otolaryngol Head Neck Surg.

[14] Hocevar-Boltezar I, Radsel Z, Zargi M (1997). The role of allergy in the etiopathogenesis of laryngeal mucosal lesions. Acta Otolaryngol Suppl.

[15] Altman KW (2007). Vocal fold masses. Otolaryngol Clin North Am.

[16] Shin J-E, Nam SY, Yoo SJ, Kim SY (2000). Changing trends in clinical manifestations of laryngeal tuberculosis. Laryngoscope.

[17] Wang C-C, Lin C-C, Wang C-P, Liu S-A (2007). Laryngeal tuberculosis: a review of 26 cases. Otolaryngol Head Neck Surg.

[18] Hong P, Liu CM, Nordstrom L, Lalwani AK (2014). The role of the human microbiome in otolaryngology-head and neck surgery: a contemporary review. Laryngoscope.

[19] Gong H-L, Shi Y, Zhou L, Wu C-P, Cao P-Y, Tao L, Xu C, Hou D-S, Wang Y-Z (2013). The composition of microbiome in larynx and the throat biodiversity between laryngeal squamous cell carcinoma patients and control population. PLoS One.

[20] Goleva E, Jackson LP, Harris JK, Robertson CE, Sutherland ER, Hall CF, Good JT, Gelfand EW, Martin RJ, Leung DYM (2013). The effects of airway microbiome on corticosteroid responsiveness in asthma. Am J Respir Crit Care Med.

[21] Wang T, Cai G, Qiu Y, Fei N, Zhang M, Pang X, Jia W, Cai S, Zhao L (2012). Structural segregation of gut microbiota between colorectal cancer patients and healthy volunteers. ISME J.

[22] Thompson CC, Emmel VE, Fonseca EL, Marin MA, Vicente ACP (2013). Streptococcal taxonomy based on genome sequence analyses. F1000Res.

[23] Kawamura Y, Hou X-G, Sultana F, Miura H, Ezaki T (1995). Determination of 16S rRNA sequences of Streptococcus mitis and Streptococcus gordonii and phylogenetic relationships among members of the genus Streptococcus. Int J Syst Bacteriol.

[24] Camelo-Castillo A, Benítez-Páez A, Belda-Ferre P, Cabrera-Rubio R, Mira A (2014). Streptococcus dentisani sp. nov., a novel member of the mitis group. Int J Syst Evol Microbiol.

[25] Sergeant MJ, Constantinidou C, Cogan T, Penn CW, Pallen MJ (2012). High-throughput sequencing of 16S rRNA gene amplicons: effects of extraction procedure, primer length and annealing temperature. PLoS One.

[26] Hanshew AS, Mason CJ, Raffa KF, Currie CR (2013). Minimization of chloroplast contamination in 16S rRNA gene pyrosequencing of insect herbivore bacterial communities. J Microbiol Methods.

[27] Jumpstart Consortium Human Microbiome Project Data Generation Working Group (2012). Evaluation of 16S rDNA-based community profiling for human microbiome research. PLoS One.

[28] Kumar PS, Brooker MR, Dowd SE, Camerlengo T (2011). Target region selection is a critical determinant of community fingerprints generated by 16S pyrosequencing. PLoS One.

[29] Engelbrektson A, Kunin V, Wrighton KC, Zvenigorodsky N, Chen F, Ochman H, Hugenholtz P (2010). Experimental factors affecting PCR-based estimates of microbial species richness and evenness. ISME J.

[30] Ahn J-H, Kim B-Y, Song J, Weon H-Y (2012). Effects of PCR cycle number and DNA polymerase type on the 16S rRNA gene pyrosequencing analysis of bacterial communities. J Microbiol.

[31] Sipos R, Székely AJ, Palatinszky M, Révész S, Márialigeti K, Nikolausz M (2007). Effect of primer mismatch, annealing temperature and PCR cycle number on 16S rRNA gene-targetting bacterial community analysis. FEMS Microbiol Ecol.

[32] Jewell KA (2014). Characterization of the Ruminal Bacterial Microbiota in Relation to Host Parameters.

[33] Segata N, Haake SK, Mannon P, Lemon KP, Waldron L, Gevers D, Huttenhower C, Izard J (2012). Composition of the adult digestive tract bacterial microbiome based on seven mouth surfaces, tonsils, throat and stool samples. Genome Biol.

[34] Huse SM, Ye Y, Zhou Y, Fodor AA (2012). A core human microbiome as viewed through 16S rRNA sequence clusters. PLoS One.

[35] Edlund A, Yang Y, Hall AP, Guo L, Lux R, He X, Nelson KE, Nealson KH, Yooseph S, Shi W, Mclean JS (2013). An in vitro biofilm model system maintaining a highly reproducible species and metabolic diversity approaching that of the human oral microbiome. Microbiome.

[36] Shahinas D, Thornton CS, Tamber GS, Arya G, Wong A, Jamieson FB, Ma JH, Alexander DC, Low DE, Pillai DR (2013). Comparative genomic analyses of Streptococcus pseudopneumoniae provide insight into virulence and commensalism dynamics. PLoS One.

[37] Arbique JC, Poyart C, Trieu-Cuot P, Quesne G, Carvalho Mda GS, Steigerwalt AG, Morey RE, Jackson D, Davidson RJ, Facklam RR (2004). Accuracy of phenotypic and genotypic testing for identification of Streptococcus pneumoniae and description of Streptococcus pseudopneumoniae sp nov. J Clin Microbiol.

[38] Laurens C, Michon A-L, Marchandin H, Bayette J, Didelot M-N, Jean-Pierre H (2012). Clinical and antimicrobial susceptibility data of 140 Streptococcus pseudopneumoniae isolates in France. Antimicrob Agents Chemother.

[39] Keith ER, Podmore RG, Anderson TP, Murdoch DR (2006). Characteristics of Streptococcus pseudopneumoniae isolated from purulent sputum samples. J Clin Microbiol.

[40] Harf-Monteil C, Granello C, Le Brun C, Monteil H, Riegel P (2006). Incidence and pathogenic effect of Streptococcus pseudopneumoniae. J Clin Microbiol.

[41] Jensen A, Fagö-Olsen H, Sørensen CH, Kilian M (2013). Molecular mapping to species level of the tonsillar crypt microbiota associated with health and recurrent tonsillitis. PLoS One.

[42] Conteduca V, Sansonno D, Lauletta G, Russi S, Ingravallo G, Dammacco F (2013). H. pylori infection and gastric cancer: state of the art (review). Int J Oncol.

[43] Yilmaz MD, Aktepe O, Cetinkol Y, Altuntas A (2005). Does Helicobacter pylori have role in development of otitis media with effusion?. Int J Pediatr Otorhinolaryngol.

[44] Birek C, Grandhi R, McNeill K, Singer D, Ficarra G, Bowden G (1999). Detection of Helicobacter pylori in oral aphthous ulcers. J Oral Pathol Med.

[45] Burduk PK (2013). Association between infection of virulence cagA gene Helicobacter pylori and laryngeal squamous cell carcinoma. Med Sci Monit.

[46] Zhuo X-L, Wang Y, Zhuo W-L, Zhang X-Y (2008). Possible association of Helicobacter pylori infection with laryngeal cancer risk: an evidence-based meta-analysis. Arch Med Res.

[47] Titiz A, Ozcakir O, Ceyhan S, Yilmaz YF, Unal A, Akyon Y (2008). The presence of Helicobacter pylori in the larynx pathologies. Auris Nasus Larynx.

[48] Tiba M, Fawaz S, Osman H (2009). Helicobacter pylori and its role in vocal folds’ minimal lesions. Clin Respir J.

[49] Masoud N, Manouchehr K, Najmeh D, Monireh H (2008). Lack of association between Helicobacter pylori and laryngeal carcinoma. Asian Pac J Cancer Prev.

[50] Aagaard K, Riehle K, Ma J, Segata N, Mistretta T-A, Coarfa C, Raza S, Rosenbaum S, Van den Veyver I, Milosavljevic A, Gevers D, Huttenhower C, Petrosino J, Versalovic J (2012). A metagenomic approach to characterization of the vaginal microbiome signature in pregnancy. PLoS One.

[51] Dickson RP, Erb-Downward JR, Freeman CM, Walker N, Scales BS, Beck JM, Martinez FJ, Curtis JL, Lama VN, Huffnagle GB (2014). Changes in the lung microbiome following lung transplantation include the emergence of two distinct Pseudomonas species with distinct clinical associations. PLoS One.

[52] Guss AM, Roeselers G, Newton ILG, Young CR, Klepac-Ceraj V, Lory S, Cavanaugh CM (2011). Phylogenetic and metabolic diversity of bacteria associated with cystic fibrosis. ISME J.

[53] The Human Microbiome Project Consortium (2012). A framework for human microbiome research. Nature.

[54] Schloss PD, Westcott SL, Ryabin T, Hall JR, Hartmann M, Hollister EB, Lesniewski RA, Oakley BB, Parks DH, Robinson CJ, Sahl JW, Stres B, Thallinger GG, Van Horn DJ, Weber CF (2009). Introducing mothur: open-source, platform-independent, community-supported software for describing and comparing microbial communities. Appl Environ Microbiol.

[55] Schloss PD, Gevers D, Westcott SL (2011). Reducing the effects of PCR amplification and sequencing artifacts on 16S rRNA-based studies. PLoS One.

[56] Quince C, Lanzen A, Davenport RJ, Turnbaugh PJ (2011). Removing noise from pyrosequenced amplicons. BMC Bioinformatics.

[57] Pruesse E, Quast C, Knittel K, Fuchs BM, Ludwig W, Peplies J, Glöckner FO (2007). SILVA: a comprehensive online resource for quality checked and aligned ribosomal RNA sequence data compatible with ARB. Nucleic Acids Res.

[58] Edgar RC, Haas BJ, Clemente JC, Quince C, Knight R (2011). UCHIME improves sensitivity and speed of chimera detection. Bioinformatics.

[59] Cole JR, Wang Q, Fish JA, Chai B, Mcgarrell DM, Sun Y, Brown CT, Porras-Alfaro A, Kuske CR, Tiedje JM (2014). Ribosomal Database Project: data and tools for high throughput rRNA analysis. Nucleic Acids Res.

[60] The Human Microbiome Project Consortium (2012). Structure, function and diversity of the healthy human microbiome. Nature.

[61] Scholz CFP, Poulsen K, Kilian M (2012). Novel molecular method for identification of Streptococcus pneumoniae applicable to clinical microbiology and 16S rRNA sequence-based microbiome studies. J Clin Microbiol.

[62] Tamura K, Stecher G, Peterson D, Filipski A, Kumar S (2013). MEGA6: Molecular Evolutionary Genetics Analysis Version 6.0. Mol Biol Evol.

[63] Wang Q, Garrity GM, Tiedje JM, Cole JR (2007). Naive Bayesian classifier for rapid assignment of rRNA sequences into the new bacterial taxonomy. Appl Environ Microbiol.

